# Proteomic profiling of endorepellin angiostatic activity on human endothelial cells

**DOI:** 10.1186/1477-5956-6-7

**Published:** 2008-02-12

**Authors:** Jason J Zoeller, Renato V Iozzo

**Affiliations:** 1 Department of Pathology, Anatomy and Cell Biology, and the Cancer Cell Biology and Signalling Program, Kimmel Cancer Center, 1020 Locust Street, Room 249 JAH, Thomas Jefferson University, Philadelphia, PA, 19107, USA

## Abstract

**Background:**

Endorepellin, the C-terminal domain V of the heparan sulfate proteoglycan perlecan, exhibits powerful and targeted anti-angiogenic activity on endothelial cells. To identify proteins involved with endorepellin anti-angiogenic action, we performed an extensive comparative proteomic analysis between vehicle- and endorepellin-treated human endothelial cells.

**Results:**

Proteomic analysis of endorepellin influence on human umbilical vein endothelial cells identified five differentially expressed proteins, three of which (β-actin, calreticulin, and chaperonin/Hsp60) were down-regulated and two of which (vimentin and the β subunit of prolyl 4-hydroxylase also known as protein disulfide isomerase) were up-regulated in response to endorepellin treatment—and associated with a fold change (endorepellin/control) ≤ 0.75 and ≥ 2.00, and a statistically significant p-value as determined by Student's *t *test.

**Conclusion:**

The proteins identified represent potential target areas involved with endorepellin anti-angiogenic mechanism of action. Further elucidation as such will ultimately provide useful in utilizing endorepellin as an anti-angiogenic therapy in humans.

## Background

Perlecan is a modular proteoglycan which is strategically located in basement membranes and cell surfaces and is capable of regulating the bioactivity of various growth factors and key receptors involved in angiogenesis and growth control [1-11]. In general, perlecan, as a whole molecule, supports vascular growth *in vivo *[12-15] and is pro-angiogenic both *in vitro *and *in vivo *[16-20]. In agreement with these studies, the anti-angiogenic fragment of antithrombin down-regulates the expression of perlecan in endothelial cells [21,22]. In contrast, the C-terminal module of perlecan, named endorepellin to designate its anti-endothelial activity, is anti-angiogenic in a dominant negative fashion [23,24]. Our previous studies have shown that endorepellin, by uniquely interacting with the α2β1 integrin, a key receptor in the angiogenesis process [25-29], signals endothelial cells to halt their migratory and capillary morphogenesis ability [24,30]. We have discovered that endorepellin specifically targets the tumor endothelium, inhibiting tumor blood vessel development and consequently tumor growth [31]. The anti-angiogenic signalling network evoked by a short exposure of endothelial cells to recombinant human endorepellin has been partially characterized at the molecular level. Following a specific interaction with the α2β1 integrin, endorepellin evokes a signalling cascade that includes activation of cAMP-dependent protein kinase A, sustained activation of focal adhesion kinase and transient increases in the phosphorylation of p38 MAPK and Hsp27 [24]. This signalling culminates into the marked disassembly of the actin cytoskeleton and focal adhesion complexes, resulting in the inhibition of endothelial cell migration [24].

The present study was designed to identify target genes affected by treatment of primary endothelial cell cultures with endorepellin. Our ultimate goal was to uncover how the endothelial cell global proteome is reprogrammed to counteract endorepellin-evoked angiostatic signals. Protein profile analysis of vehicle- versus endorepellin-treated endothelial cells identified five differentially expressed proteins of interest, of which three were down-regulated and two were up-regulated, in response to endorepellin treatment. The proteins identified, which include proteins already known to be involved in the angiogenic process such as calreticulin/vasostatin, actin and protein disulfide isomerase, represent potential targets involved in endorepellin anti-angiogenic mechanism of action.

## Results and Discussion

**Figure 1:**
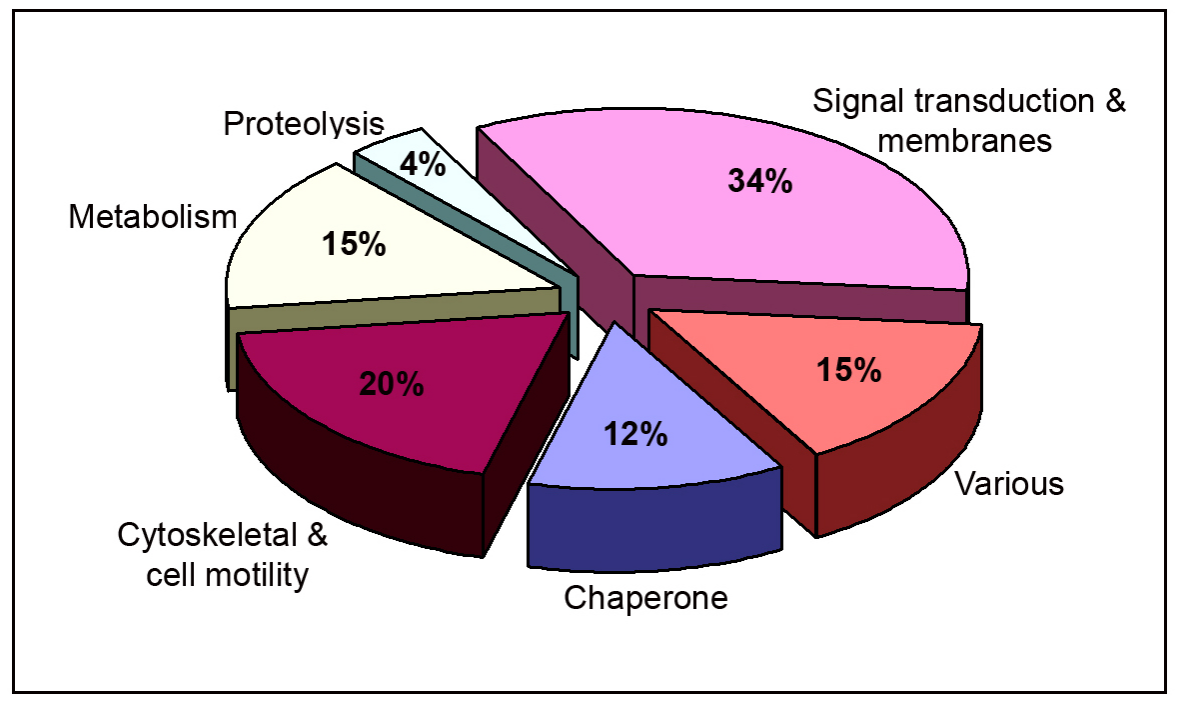
**Classification of the 106 protein spots positively identified during the HUVEC proteome analysis**. All proteins identified were categorized into one of six functional groups. Please see Additional file 1 – Description and summary of the proteins identified in the comparative proteome's of HUVEC control and endorepellin-treated, for a complete listing and further information regarding the proteins corresponding to each group.

To identify proteins involved with endorepellin angiostatic action, we performed an extensive comparative proteomic analysis between vehicle- and endorepellin-treated endothelial cell proteomes. Each recombinant endorepellin batch was tested for positive biological activity utilizing a highly sensitive actin disassembly assay [24], and protein response was analyzed following 2 h, 500 nM endorepellin treatment. We selected the 2 hour treatment period for this particular study to reflect global changes in endothelial cell protein synthesis affected downstream in the endorepellin signaling pathway. One hundred and six protein spots were positively identified in our HUVEC 2-D protein profiles. All identified proteins were categorized into one of six functional groups (Figure 1 and Additional file 1) with the majority of proteins functionally classified as signal transduction and membrane-associated (34%) or cytoskeletal and cell motility-related (20%). The remaining were classified as metabolic or various proteins (15%, each) and as chaperone or proteolytic proteins (12 and 4%, respectively).

**Figure 2:**
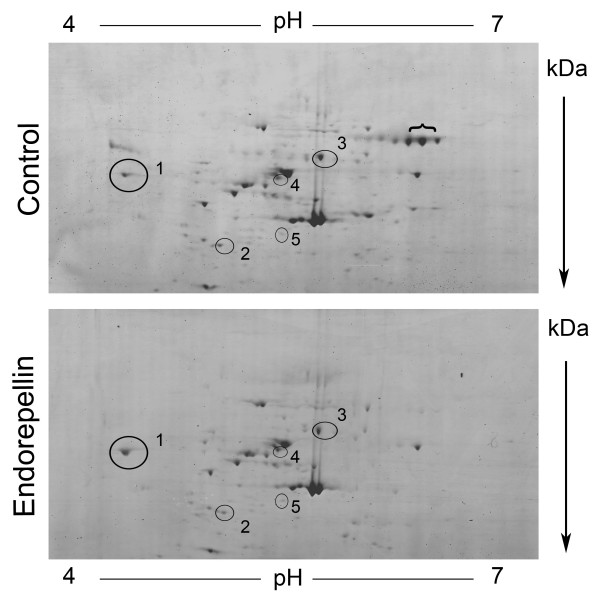
**Comparative proteomic analysis of vehicle- and endorepellin-treated human endothelial cells**. Representative 2-D protein profiles from vehicle- and endorepellin-treated endothelial cells are depicted with protein separation according to isoelectric point (pI, x-axis) and molecular weight (kDa, y-axis). Encircled protein spots correspond to the five proteins differentially expressed in response to endorepellin treatment. Compare calreticulin (1), β-actin (2), chaperonin/Hsp60 (3), vimentin (4) and prolyl 4-hydorxylase, β subunit (5), spot intensities in the two sets of samples. The spots under the bracket represent serum albumin by LC-MS/MS.

We focused our analysis on the majority of endothelial cell proteins within the pI 4 – 7 range and utilized 8–18% gradient gels to achieve separation of the proteins in the second dimension. Protein spots were detected by the highly sensitive colloidal blue stain which can detect < 10 ng of protein. Comparative proteomic analysis identified five distinct proteins with differential expression levels in response to endorepellin treatment (Figure 2 and Table 1). All five spots were associated with a fold change (endorepellin/control) ≤ 0.75 and ≥ 2.00, and a p-value ~< 0.1 (considered statistically significant). Fold changes were calculated by the formula 10^ (log-scale mean Endorepellin – log-scale mean Control). Statistical analysis of protein expression data was completed through missing value imputation via K-nearest neighbors (KNN, K = 10) analysis accounting for gel-to-gel variability [32,33], followed by log-transformation of the imputed data and comparison of experimental data with statistical validation by Student's *t *test.

**Table 1:** Differential expression of endothelial cell proteins evoked by exposure to recombinant human endorepellin

***Spot number (see Fig. 2)***	***Protein ID***	***Gene name***	***NCBI Accession Number***	***Mr (kDa)***	***pI***	***Response to endorepellin (mean fold change)***	***p-value***	***Reported functions*^a^**
**1**	Calreticulin	CALR	4757900	48	4.14	↓ Downregulated, 0.642	0.0881	Molecular calcium binding and chaperone protein
**2**	β-actin	ACTB	4501885	42	5.18	↓ Downregulated, 0.403	0.0023	Cellular structure, motility and integrity
**3**	Chaperonin/Hsp60	HSPD1	31542947	61	5.59	↓ Downregulated, 0.714	0.1262	Mitochondrial protein assembly, prevention of misfolding, produced under stress within the mitochondrial matrix
**4**	Vimentin	VIM	4507895	54	4.91	↑ Upregulated, 3.178	0.0276	Class III intermediate filament, cytoskeletal crosstalk, intracellular communication, influence cell shape, adhesion and migration, signalling events
**5**	Prolyl 4-hydroxylase, β subunit	P4HB	20070125	57	4.61	↑ Absent from Control^b^	-	Key subunit of prolyl 4-hydroxylase enzyme, identical to protein disulfide isomerase

^a^As reported in the protein database on the ExPASy web server and in the NCBI Entrez Protein database.

^b^Trace amounts of this enzyme were found by immunoblotting (see Figure 3 E)

In response to endorepellin treatment, three spots were significantly down-regulated (fold change ≤ 0.75), one spot was significantly up-regulated (fold change ≥ 2.00) and one spot was completely absent from the control (Table 1). The five spots were identified by Electrospray LC-MS/MS analysis as calreticulin, β-actin, chaperonin/Hsp60 (Figure 2, spot numbers 1–3, respectively), vimentin (spot no. 4) and prolyl 4-hydroxylase, β subunit (spot no. 5). Notably, all five proteins identified have been functionally linked to the α2β1 integrin and/or associated with angiogenesis. Therefore each protein may contribute to the molecular basis of endorepellin anti-angiogenic mechanism of action on the endothelium.

### β-actin

Endothelial cell migration and adhesion depend on cytoskeletal remodeling mediated by actin dynamics [34,35]. Cell migration has been associated with β-actin mRNA localization and increased actin synthesis [36,37]. Angiogenesis inhibitors, such as endostatin and endorepellin, target endothelial cell migration by interfering with actin organization[24,38-40].

**Figure 3:**
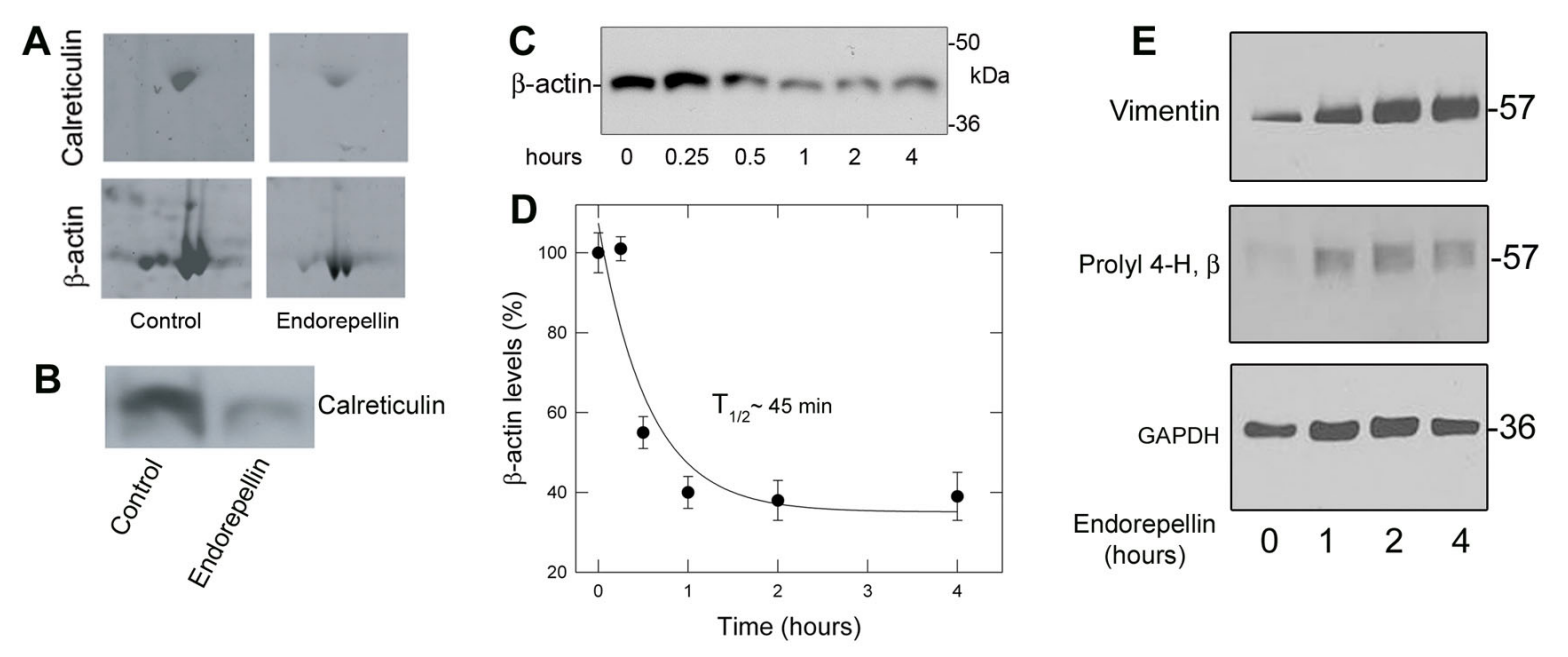
**Immunoblotting supports the proteomic analysis and indicates relative changes of various endothelial cell proteins evoked by endorepellin treatment**. (A) Close up view of two representative 2-D gels, depicting the decline in calreticulin and β-actin levels. Notice that the major actin isoforms also show reduced staining intensity in response to endorepellin treatment. (B) Immunoblotting of control and endorepellin-treated endothelial cell lysates using an antibody against human calreticulin. Equal amounts of total proteins were loaded. (C) Immunoblot analysis of β-actin levels following treatment with ~200 nM endorepellin for the designated time intervals. (D) The kinetic data for β-actin expression levels were derived from immunoblotting data as presented in C. Essentially HUVEC β-actin expression levels were examined by Western blot following exposure to endorepellin for the indicated time points. β-actin levels were quantified over similar amounts of protein loading and graphed as percent β-actin levels (based upon time point 0) versus exposure to endorepellin treatment (hours). The data were graphed in SigmaPlot v9 and fit by non-linear regression analysis. Each data point represents the mean ± S.E.M. from three experiments. The T1/2 of ~45 min. represents the time to reduce β-actin levels by 50% as compared to the control. (E) Immunoblot analysis of vimentin and prolyl 4-hydroxylase, β subunit protein levels following endorepellin treatment for the indicated time points. GAPDH is shown as loading control. All verification experiments presented in panels C-E utilized new samples of endorepellin-treated HUVEC lysates, separate from those used in proteomic analysis.

Endorepellin was found to decrease the protein levels of endothelial cell actin (Figures 2 and 3A). In additional experiments, the continuous exposure to endorepellin (200 nM) resulted in a decline of endothelial cell β-actin levels with a T_1/2 _of ~45 minutes (Figure 3C and 3D). The observed changes in actin may reflect endorepellin action through the α2β1 integrin [24]. Previously, we identified α2β1 integrin as the major receptor for endorepellin on endothelial cells, and associated the interaction with promoting endothelial cell disassembly of the actin cytoskeleton and focal adhesion complexes. The observed decrease in actin may be directly related to the breakdown of the cellular actin network which would interfere with endothelial cell migration and capillary morphogenesis.

### Calreticulin

One of the key proteins identified in our proteomics analysis and validated by immunoblotting, was calreticulin (Figure 3A and 3B), a multifunctional and widespread calcium-binding protein found in the endoplasmic reticulum, but also at the cell surface and as a secreted form [41]. Additionally, vasostatin, a fragment of calreticulin, inhibits angiogenesis and tumor growth [42,43]. The localization of calreticulin in other cellular compartments has prompted reconsideration of this protein as a mediator of a broader array of cellular functions.

Notably, calreticulin is essential for integrin-mediated calcium signalling and adhesion [44], and interacts with α2β1 on the surface of platelets [45], Jurkat and PC3 cells [46,47]. Calreticulin can regulate adhesion by a number of mechanisms. For example, calreticulin modulates the affinity of α2β1 integrin for its ligands via transient interactions with the cytoplasmic domain of the integrin α2 subunit [48]. The transient interaction is believed to function during the formation of a primary pro-adhesive cellular complex but may not be required during the later establishment of a full focal adhesion [46,49]. Consistent with this, calreticulin-deficient cells have impaired cell adhesion [44], whereas cells overexpressing calreticulin have increased levels of vinculin and are consequently more adhesive [50]. Thus, a possible contributing factor to the anti-adhesive properties of endorepellin would be reducing intracellular calreticulin levels.

Calreticulin regulation by endorepellin must also be considered beyond the subcellular levels and extend to the cell surface. Cell surface calreticulin, in contrast to intracellular calreticulin, actually counters cell adhesion. The extracellular matrix thrombospondin proteins function through cell surface calreticulin to promote focal adhesion disassembly and ultimately favor cell migration [51-53]. Our proteomics study was not capable of distinguishing between cell surface and intracellular calreticulin. Additionally an endorepellin—cell surface-calreticulin functional and cooperative interaction has not been investigated. The potential for the observed endorepellin-induced downregulation of calreticulin to represent changes in either cell surface or intracellular calreticulin levels alone versus overall total levels is intriguing to consider within the context of endothelial cell adhesion, migration and endorepellin function.

### Chaperonin/Hsp60

We found that endorepellin treatment reduced endothelial expression levels of chaperonin/Hsp60. The heat shock protein family member chaperonin (heat shock protein 60-kDa or mitochondrial matrix protein P1) functions primarily in cooperation with Hsp10 to aide in mitochondrial protein folding, unfolding and degradation events [54]. Numerous other functions have been attributed to chaperonin/Hsp60 outside of its role as a classical heat shock protein, including α3β1-specific integrin activation and influencing apoptotic cell death [54,55].

While the observed down-regulation could presumably disturb the endothelial cell mitochondrial stress response, perhaps the decreased expression may promote apoptosis in these cells. Indeed, attenuated expression of Hsp60 by antisense mediated knockdown in cardiac myocytes has been associated with promoting apoptosis [56]. Additionally the PPARγ agonist, PGJ_2_(15-deoxy-Δ^12–14^-prostaglandin J_2_) has pro-apoptotic effects on human vascular endothelial cells that has been associated with a decrease in Hsp60 expression [57]. Intriguingly, endothelial cells during apoptotic death release into their media a fragment of endorepellin which could act in a paracrine fashion on adjacent cells [58]. The exact apoptotic role of chaperonin/Hsp60 may be a complex and variable one [56,59,60], but it is intriguing to consider in light of the anti-angiogenic influence of endorepellin on the endothelium.

### Vimentin

Vimentin is a type-III intermediate filament of the cellular cytoskeleton. Intermediate filaments form a cage-like network throughout the entire cell, which links intermediate filaments to cytoskeletal crosstalk among microfilaments, microtubules, and mediates signaling events from one region of the cell to another [61]. Vimentin intermediate filaments are capable of influencing cell shape, cell adhesion and migration and cell signaling events.

Notably, the α2β1 integrin interacts with vimentin and co-localizes with the integrin in endothelial cell focal adhesions and contributes to the vimentin-associated matrix adhesion structures identified in endothelial cells [62,63]. Given these roles, a decreased expression of vimentin protein levels might be predicted to support anti-angiogenic action. Unexpectedly, our studies found that endorepellin increased vimentin protein levels (Table I and Figure 3E). Increased expression of vimentin may negatively contribute to the dynamics of cytoskeletal rearrangement induced by endorepellin action or may represent an increase in vimentin cleavage products as a result of apoptosis. Indeed, vimentin is a substrate for caspase cleavage. Cleavage produces vimentin fragments that inhibit continued intermediate filament assembly, and results in the breakdown of the intermediate filament cytoskeletal network—hallmarks of apoptotic cell death [64].

### Prolyl 4-hydroxylase, β subunit

Collagen biosynthesis requires the enzyme prolyl 4-hydroxylase, which catalyzes the formation of 4-hydroxyproline [65]. Hydroxylation at these residues is a key factor in facilitating collagen triple helix assembly and stability. Prolyl 4-hydroxylase is a tetramer composed of two α and two β subunits, with the β subunit being identical to protein disulfide isomerase [66,67]. Protein disulfide isomerase possesses an enzymatic role, catalyzing protein disulfide bond formation and a chaperone role, promoting proper protein folding [68-70]. In the proteomics analysis, endothelial cells did not show any detectable levels of the enzyme by using Colloidal blue in contrast to the endorepellin-treated cells (Figure 2). However by immunoblotting using an antibody specific for the β subunit we were able to detect low levels of the enzyme and these levels were markedly upregulated by endorepellin (Figure 3E). The immunoblotting data further indicate a sustained induction of the enzyme with kinetics similar to those shown for vimentin (Figure 3E).

We believe the observed increase in the P4-H β subunit may be related to the protein's inherent dual roles. Enzymatically active prolyl 4-hydroxylase requires the β subunit for proper tetramer formation, folding and to prevent aggregation of the α subunits [71]. The increase in β subunits could potentially promote the formation of the active enzyme, essentially favor collagen biosynthesis and eventual deposition into the extracellular matrix. Endorepellin may induce prolyl 4-hydroxylase levels as a means to support extracellular matrix assembly and counter angiogenesis since the matrix serves as a barrier to migrating endothelial cells.

The β subunit in the α_2_β_2 _tetramer as well as monomer form is equivalent to protein disulfide isomerase and both possess disulfide isomerase activity. The endorepellin-evoked increase in β subunit monomers may actually be an increase in the chaperone functioning protein disulfide isomerase. Since proper protein folding by chaperones is essential to protein synthesis, we believe that the endorepellin-evoked increase in β subunits/protein disulfide isomerase levels may link anti-angiogenic endorepellin to interfering with protein synthesis. Of note, tumstatin, the anti-angiogenic fragment of type IV collagen, inhibits endothelial cell protein synthesis and results in apoptotic cell death [72,73].

## Conclusion

Comparative assessment of vehicle- and endorepellin-treated primary endothelial cells identified five proteins with differential expression patterns in response to endorepellin. The identification of these endothelial cell proteins, which are directly or indirectly related to the α2β1 integrin and angiogenic processes, provides new insight into the molecular and cellular biology of endorepellin anti-angiogenic network by representing potential target areas involved with endorepellin action. Understanding the basis for endorepellin targeted action on the endothelium, by identifying new proteins and links to new pathways, may ultimately provide useful in utilizing endorepellin as an anti-angiogenic therapy in humans.

## Methods

### Protein extraction from human umbilical vein endothelial cells and quantification

2 × 10^6 ^human umbilical vein endothelial cells (VEC Technologies, Inc.) were cultured to confluence in M131 endothelial cell growth factor-supplemented media. Endothelial cells were either treated with 500 nM endorepellin-supplemented media (experimental, n = 5) or PBS-supplemented media (control, n = 5) for 2 hours. Cell cultures were gently washed in Hank's balanced salt solution (with Ca^+2^/Mg^+2^) and protein was extracted by total cell lysis in 4% CHAPS; 8 M urea lysis buffer. Cell lysates were collected via gentle scraping, centrifuged at 15,000 RPM for 10 minutes at 4°C, and followed by mild sonication over ice. Protein was quantified using the 2-D Quant Kit (GE Healthcare), collected and stored at -80°C.

### Two-dimensional (2-D) gel electrophoresis

Forty μg of total protein lysate from control (n = 5) and endorepellin-treated (n = 5) endothelial cells were each suspended in lysis buffer (4% CHAPS; 8 M urea), 1 M DTT, IPG buffer pH 4–7 and a trace of bromophenol blue at a final volume of 350 μl. Sample mixtures were centrifuged at 14,000 RPM for 15 minutes. Protein samples were separated in the first dimension on 18 cm IPG strips pH 4–7 using an IPG phor (GE Healthcare) with active rehydration at 30 V for 12 h followed by focusing at 500 V for 1 h, 1000 V for 1 h and 8000 V for 6 h. Prior to protein separation in the second dimension, the IPG strips were equilibrated for fifteen minutes with gentle agitation at room temperature in equilibration buffer (20% glycerol, 2% SDS, 0.375 M Tris/HCl pH 8.8, 6 mol/L urea) containing 13 mM DTT followed by equilibration buffer containing 2.5% iodoacetamide. IPG strips were then directly applied to 8–18% SDS Tris-Glycine gels (Jule Inc. Biotechnologies), and protein separation in the second dimension was performed using the DALT6 platform (GE Healthcare) at 2 W/gel for thirty minutes, followed by 20 W/gel until completion. 2-D gels were stained with the protein sensitive Colloidal Blue Staining Kit according to the manufacturer's protocol and stored in ultrapure water at 4°C (Invitrogen Life Technologies). Each gel image was recorded by the Typhoon fluorescent imager and the Image Quant software program (GE Healthcare).

### Protein profile analysis and mass spectrometry

To compare proteins differentially expressed in control and endorepellin-treated samples, each of the ten gel 2-D images was analyzed in the DIA program of the DeCyder v5.01 software package (GE Healthcare). The DIA program compares protein expression profiles by assessing and quantifying the intensity of the protein spots identified in each gel. Each of the ten DIA analysis files were collected and analyzed together in the DeCyder BVA program. The BVA program identified reproducible spots of interest in control and endorepellin-treated HUVEC proteomes. These protein spots of interest were extracted from the gel and trypsin digested by the Ettan Dalt spot handling work station (GE Healthcare) in preparation for protein identification by Electrospray LC-MS/MS analysis (Finnigan Proteome X LTQ). Sequence analysis was performed with the BioWorks Browser v3.2 software package using, XCorr, ΔCn (>0.1), RSp (<4) and probability values (<1), as filters for positive protein identification.

### Differential protein expression data analysis and statistical validation

Statistical analysis of protein expression data was completed through missing value imputation via K-nearest neighbors (KNN, K = 10) analysis as previously described [32,33], followed by log-transformation of the imputed data and comparison of control and endorepellin-treated values with statistical validation by Student's *t *test (p-value ~< 0.1, considered statistically significant). Fold changes were calculated by the formula 10^(log-scale mean endorepellin – log-scale mean control). Proteins were categorized according to protein expression, down-regulated (fold change ≤ 0.75) or up-regulated (fold change ≥ 2), in response to endorepellin treatment.

### Immunoblotting endothelial cell protein lysates

Endothelial cells were endorepellin treated as described above. Cell lysates were collected in RIPA buffer, separated by SDS-PAGE and transferred to Nitrocellulose (Biorad). Immunoblotting was performed with polyclonal anti-calreticulin (Stressgen, SPA-600); monoclonal anti-β-actin (Sigma, A 5316); monoclonal anti-vimentin (Sigma, V 6630) and monoclonal anti-prolyl-4-hydroxylase β (ImmunO, 63164). Primary antibodies were detected with donkey anti-rabbit HRP (GE Healthcare) or goat anti-mouse HRP (Pierce) secondary antibodies, and developed by ECL (Pierce).

## Competing interests

The author(s) declare that they have no competing interests.

## Authors' contributions

JJZ contributed in the execution of all the experimental data and drafting of the manuscript. RVI was responsible for designing the experimental strategy and writing the manuscript. JJZ and RVI read and approved the final manuscript.

## Supplementary Material

Additional file 1 **Description and summary of the proteins identified in the comparative proteome analysis of vehicle- and endorepellin-treated human endothelial cells**. The following table represents the 106 proteins identified, categorized according to the functional designations used in Figure 1. A brief summary of protein function is described as reported in the protein database on the ExPASy web server and or in the NCBI Entrez Protein database. Please refer to the NCBI Accession numbers presented within the table for additional specific information. Proteins highlighted in blue signify identified proteins with differential expression in response to endorepellin treatment- associated with a fold change ≤ 0.75 and ≥ 2.00, and a statistically significant p-value ~<0.1 as determined by Student's *t *test. Click here for file
