# Supplementary material for: Proteomic profiling of endorepellin angiostatic activity on human endothelial cells
Source: Proteome Sci. 2008 Feb 12;6:7. doi: 10.1186/1477-5956-6-7 (PMC2275231; doi:10.1186/1477-5956-6-7)
Supplement: Additional file 1 — Description and summary of the proteins identified in the comparative proteome analysis of vehicle- and endorepellin-treated human endothelial cells. The following table represents the 106 proteins identified, categorized according to the functional designations used in Figure 1. A brief summary of protein function is described as reported in the protein database on the ExPASy web server and or in the NCBI Entrez Protein database. Please refer to the NCBI Accession numbers presented within the table for additional specific information. Proteins highlighted in blue signify identified proteins with differential expression in response to endorepellin treatment- associated with a fold change ≤ 0.75 and ≥ 2.00, and a statistically significant p-value ~<0.1 as determined by Student's t test. [file 1477-5956-6-7-S1.DOC]

Additional File 1.

**Description and summary of the proteins identified in the comparative proteome analysis of vehicle- and endorepellin-treated human endothelial cells.** The following table represents the 106 proteins identified, categorized according to the functional designations used in Figure 1. A brief summary of protein function is described as reported in the protein database on the ExPASy web server and or in the NCBI Entrez Protein database. Please refer to the NCBI Accession numbers presented within the table for additional specific information. Proteins highlighted in blue signify identified proteins with differential expression in response to endorepellin treatment- associated with a fold change ≤ 0.75 and ≥ 2.00, and a statistically significant p-value ~<0.1 as determined by Student’s *t* test.

| ***Protein Identity*** | ***NCBI Accession***  ***No.*** | ***Function*** |
| --- | --- | --- |
| ***Chaperone* (12%)** |  |  |
| calreticulin precursor  ******fold change* (μ ER / μ Control) = 0.642 | 4757900 | Molecular calcium binding chaperone promoting folding, oligomeric assembly and quality control in the ER via the calreticulin/calnexin cycle. It regulates cell adhesion by interacting with α2β1integrin. It also interacts with thrombospondin and regulates focal adhesion disassembly. This lectin interacts transiently with almost all of the monoglucosylated glycoproteins that are synthesized in the ER. |
| cardiac calsequestrin 2 | 4557409 | Calsequestrin is a high-capacity, moderate affinity, calcium-binding protein and thus acts as an internal calcium store in muscle. |
| Chaperonin  (Hsp60/mitochondrial matrix protein P1)  ******fold change* (μ ER / μ Control) = 0.714 | 31542947 | Implicated in mitochondrial protein import and macromolecular assembly. May facilitate the correct folding of imported proteins. May also prevent misfolding and promote the refolding and proper assembly of unfolded polypeptides generated under stress conditions in the mitochondrial matrix. |
| chaperonin containing TCP1, subunit 5 (epsilon) | 24307939 | Molecular chaperone; assists the folding of proteins upon ATP hydrolysis. Known to play a role, in vitro, in the folding of actin and tubulin. |
| glucose regulated protein, 58kDa  (protein disulfide isomerase-associated 3 precursor) | 21361657 | A protein of the endoplasmic reticulum that interacts with lectin chaperones calreticulin and calnexin to modulate folding of newly synthesized glycoproteins. The protein has protein disulfide isomerase activity. It is thought that complexes of lectins and  this protein mediate protein folding by promoting formation of  disulfide bonds in their glycoprotein substrates. |
| heat shock 27kDa protein 1 | 4504517 | Involved in stress resistance and actin organization. |
| heat shock 70kD protein binding protein | 19923193 | An adaptor protein that mediates the association of the heat shock proteins HSP70 and HSP90. |
| heat shock 70kDa protein 5 (glucose-regulated protein, 78kDa)  heat shock 70kDa proteins | 16507237 | GRP78 probably plays a role in facilitating the assembly of multimeric protein complexes inside the ER. Hsp70s, in cooperation with other chaperones, stabilize preexistent proteins against aggregation and mediate the folding of newly translated polypeptides in the cytosol as well as within organelles. These chaperones participate in all these processes through their ability to recognize nonnative conformations of other proteins. They bind extended peptide segments with a net hydrophobic character exposed by polypeptides during translation and membrane translocation, or following stress-induced damage. |
| heat shock 90kDa protein 1, beta | 20149594 | Molecular chaperone. |
| prolyl 4-hydroxylase, beta subunit  ******fold change* (μ ER / μ Control) = *ABSENT* from Control. | 20070125 | This multifunctional protein catalyzes the formation, breakage and rearrangement of disulfide bonds. At the cell surface, seems to act as a reductase that cleaves disulfide bonds of proteins attached to the cell. May therefore cause structural modifications of exofacial proteins. Inside the cell, seems to form/rearrange disulfide bonds of nacent proteins. At high concentrations, functions as a chaperone that inhibits aggregation of misfolded proteins. At low concentrations, facilitates aggregation (antichaperone activity). Also acts a structural subunit of various enzymes such as prolyl 4-hydroxylase and microsomal triacylglycerol transfer protein MTTP. |
| protein disulfide isomerase-associated 6 | 5031973 | Catalyzes the rearrangement of -S-S- bonds in proteins. |
| thioredoxin domain containing 5 isoform 1 | 42794771 | Possesses thioredoxin activity. |
| tumor rejection antigen (gp96) 1 | 4507677 | Molecular chaperone that functions in the processing and transport of secreted proteins. |
| ***Cytoskeletal & Cell Motility* (20%)** |  |  |
| alpha 1 actin precursor  beta actin  ******fold change* (μ ER / μ Control) = 0.403 | 4501881  4501885 | Actins are highly conserved proteins that are involved in cell motility, structure and integrity. Alpha actins are a major constituent of the contractile apparatus. Beta actin is one of six different actin isoforms which have been identified. |
| cardiac leiomodin, similar to | 51492827 | Cytoskeleton; tropomyosin binding, predicted. |
| chondroadherin precursor | 4502799 | Promotes attachment of chondrocytes, fibroblasts, and osteoblasts. This binding is mediated (at least for chondrocytes and fibroblasts) by the integrin alpha(2)beta(1). May play an important role in the regulation of chondrocyte growth and proliferation. |
| cortactin isoform a | 20357552 | Has two roles: (1) regulating the interactions between components of adherens-type junctions and (2) organizing the cytoskeleton and cell adhesion structures of epithelia and carcinoma cells. During apoptosis, the encoded protein is degraded in a caspase-dependent manner. The aberrant regulation of this gene contributes to tumor cell invasion and metastasis. |
| desmin | 18105050 | Desmin are class-III intermediate filaments found in muscle cells. In adult striated muscle they form a fibrous network connecting myofibrils to each other and to the plasma membrane from the periphery of the Z-line structures. |
| dynein, cytoplasmic, intermediate polypeptide 2 | 24307879 | The intermediate chains seem to help dynein bind to dynactin 150 kDa component. May play a role in mediating the interaction of cytoplasmic dynein with membranous organelles and kinetochores. |
| F-actin capping protein alpha-1 subunit | 5453597 | F-actin capping proteins bind in a Ca(2+)-independent manner to the fast growing ends of actin filaments (barbed end) thereby blocking the exchange of subunits at these ends. Unlike other capping proteins (such as gelsolin and severin), these proteins do not sever actin filaments. |
| internexin neuronal intermediate filament protein, alpha | 14249342 | Class-IV neuronal intermediate filament that is able to self-assemble. |
| lamin B2 | 27436951 | Lamins are components of the nuclear lamina, a fibrous layer on the nucleoplasmic side of the inner nuclear membrane, which is thought to provide a framework for the nuclear envelope and may also interact with chromatin. |
| myosin, heavy polypeptide 9, non-muscle | 12667788 | Cellular myosin that appears to play a role in cytokinesis, cell shape, and specialized functions such as secretion and capping. |
| myristoylated alanine-rich protein kinase C substrate (MARCKS) | 11125772 | MARCKS is the most prominent cellular substrate for protein kinase C. This protein binds calmodulin, actin, and synapsin. MARCKS is a filamentous (F) actin cross-linking protein. The protein is thought to be involved in cell motility, phagocytosis, membrane trafficking and mitogenesis. |
| ninein isoform 4 | 33946313 | Centrosomal protein required in the positioning and anchorage of the microtubule minus-end in epithelial cells. May also act as a centrosome maturation factor. May play a role in microtubule nucleation. Overexpression does not perturb nucleation or elongation of microtubules but suppresses release of microtubules. |
| nuclear distribution gene C homolog (nudC) | 5729953 | Plays a role in neurogenesis and neuronal migration. Necessary for correct formation of mitotic spindles and chromosome separation during mitosis. Necessary for cytokinesis and cell proliferation. Colocalizes with tubulin and dynein and with the microtubule organizing center. |
| peripherin | 21264345 | Peripherin are class-III neuronal intermediate filament proteins. |
| pleckstrin homology domain containing,  family H (with MyTH4 domain) member 2 | 45120121 | Cytoskeleton; actin binding; ATP binding; motor activity, predicted. |
| smoothelin isoform c | 19913394 | Structural protein, it associates with stress fibers and constitutes part of the cytoskeleton. |
| tubulin alpha 6  tubulin, alpha-like 3  tubulin, betas | 14389309  13376181  29788768 | Tubulin is the major constituent of microtubules. It binds two moles of GTP, one at an exchangeable site on the beta chain and one at a nonexchangeable site on the alpha-chain. |
| vimentin  ******fold change* (μ ER / μ Control) = 3.178 | 4507895 | Vimentins are class-III intermediate filaments. |
| ***Metabolic* (15%)** |  |  |
| ATPase, H+/K+ transporting, nongastric, alpha polypeptide; ATPase, Na+K+ transporting, alpha-1 polypeptide-like; ATPase, Na+/K+ transporting, alpha polypeptide-like 1 | 10280618 | Catalyzes the hydrolysis of ATP coupled with the exchange of H(+) and K(+) ions across the plasma membrane. Responsible for potassium absorption in various tissues. CATALYTIC ACTIVITY: ATP + H_2_O + H^+^(In) + K^+^(Out) = ADP + phosphate + H^+^(Out) + K^+^(In). |
| ATP synthase, H+ transporting, mitochondrial F1 complex,  beta subunit precursor | 32189394 | Mitochondrial ATP synthase catalyzes ATP synthesis, utilizing an electrochemical gradient of protons across the inner membrane  during oxidative phosphorylation. ATP synthase is composed of two linked multi-subunit complexes: the soluble catalytic core, F1, and the membrane-spanning component, Fo, comprising the proton channel. The catalytic portion of mitochondrial ATP synthase consists of 5 different subunits (alpha, beta, gamma, delta, and epsilon) assembled with a stoichiometry of 3 alpha, 3 beta, and a single representative of the other 3. The proton channel consists of three main subunits (a, b, c). |
| enolase 1 (Alpha-enolase) | 4503571 | Multifunctional enzyme that, as well as its role in glycolysis, plays a part in various processes such as growth control, hypoxia tolerance and allergic responses. May also function in the intravascular and pericellular fibrinolytic system due to its ability to serve as a receptor and activator of plasminogen on the cell surface of several cell-types such as leukocytes and neurons. CATALYTIC ACTIVITY: 2-phospho-D-glycerate = phosphoenolpyruvate + H_2_O. |
| enolase 2 (Gamma-enolase) | 5803011 | Has neurotrophic and neuroprotective properties on a broad spectrum of central nervous system (CNS) neurons. Binds, in a calcium-dependent manner, to cultured neocortical neurons and promotes cell survival. CATALYTIC ACTIVITY: 2-phospho-D-glycerate = phosphoenolpyruvate + H_2_O. |
| enolase 3 (Beta-enolase) | 16554592 | Appears to have a function in striated muscle development and regeneration. [CATALYTIC ACTIVITY](http://www.expasy.org/sprot/userman.html#CC_line): 2-phospho-D-glycerate = phosphoenolpyruvate + H_2_O. |
| glutathione transferase | 4504183 | Glutathione S-transferases (GSTs) are a family of enzymes that play an important role in detoxification by catalyzing the conjugation of many hydrophobic and electrophilic compounds with reduced glutathione. |
| glyceraldehyde-3-phosphate dehydrogenase | 7669492 | Glyceraldehyde-3-phosphate dehydrogenase catalyzes an important energy-yielding step in carbohydrate metabolism, the reversible oxidative phosphorylation of glyceraldehyde-3-phosphate in the presence of inorganic phosphate and nicotinamide adenine dinucleotide (NAD). The enzyme exists as a tetramer of identical chains. CATALYTIC ACTIVITY: D-glyceraldehyde 3-phosphate + phosphate + NAD^+^ = 3-phospho-D-glyceroyl phosphate + NADH. |
| mitochondrial malate dehydrogenase precursor | 21735621 | Malate dehydrogenase catalyzes the reversible oxidation of malate to oxaloacetate, utilizing the NAD/NADH cofactor system in the citric acid cycle. The protein encoded by this gene is localized to the mitochondria and may play pivotal roles in the malate-aspartate shuttle that operates in the metabolic coordination between cytosol and mitochondria. CATALYTIC ACTIVITY: (S)-malate + NAD^+^ = oxaloacetate + NADH. |
| mitochondrial short-chain enoyl-coenzyme A hydratase 1 precursor | 12707570 | The protein functions in the second step of the mitochondrial fatty acid beta-oxidation pathway. It catalyzes the hydration of 2-trans-enoyl-coenzyme A (CoA) intermediates to L-3-hydroxyacyl-CoAs. |
| peroxiredoxin 6 | 4758638 | The protein is a member of the thiol-specific antioxidant protein family. Involved in redox regulation of the cell. Can reduce H(2)O(2) and short chain organic, fatty acid, and phospholipid hydroperoxides. May play a role in the regulation of phospholipid turnover as well as in protection against oxidative injury. CATALYTIC ACTIVITY: 2 R'-SH + ROOH = R'-S-S-R' + H_2_O + ROH. |
| serum/glucocorticoid regulated kinase 2 isoform beta | 20127541 | CATALYTIC ACTIVITY: ATP + a protein = ADP + a phosphoprotein. |
| succinate-CoA ligase, ADP-forming, beta subunit | 11321583 | Metabolism; ligase activity, predicted. |
| superoxide dismutase 1, soluble | 4507149 | Destroys radicals which are normally produced within the cells and which are toxic to biological systems. [CATALYTIC ACTIVITY](http://www.expasy.org/sprot/userman.html#CC_line): 2 superoxide + 2 H^+^ = O_2_ + H_2_O_2_. |
| surfeit 1 | 4507319 | Probably involved in the biogenesis of the COX complex. |
| ubiquinol-cytochrome c reductase core protein I | 46593007 | This is a component of the ubiquinol-cytochrome c reductase complex (complex III or cytochrome b-c1 complex), which is part of the mitochondrial respiratory chain. This protein may mediate formation of the complex between cytochromes c and c1. CATALYTIC ACTIVITY: QH_2_ + 2 ferricytochrome c = Q + 2 ferrocytochrome c. |
| vesicle amine transport protein 1 | 18379349 | Does not have alcohol dehydrogenase activity. Binds NADP and acts through a one-electron transfer process. Orthoquinones are the best substrates. May act in the detoxification of xenobiotics. |
| ***Proteolytic* (4%)** |  |  |
| proteasome beta 2 subunit | 4506195 | The proteasome is a multicatalytic proteinase complex with a highly ordered ring-shaped 20S core structure. The core structure is composed of 4 rings of 28 non-identical subunits; 2 rings are  composed of 7 alpha subunits and 2 rings are composed of 7 beta  subunits. Proteasomes are distributed throughout eukaryotic cells  at a high concentration and cleave peptides in an ATP/ubiquitin dependent process in a non-lysosomal pathway. |
| S-phase kinase-associated protein 1A isoform a | 25777711 | Essential component of the SCF (SKP1-CUL1-F-box protein) ubiquitin ligase complex, which mediates the ubiquitination of proteins involved in cell cycle progression, signal transduction and transcription. In the SCF complex, serves as an adapter that links the F-box protein to CUL1. The encoded protein also collaborates with a network of proteins to control beta-catenin levels and affects the activity level of beta-catenin dependent TCF transcription factors. Studies have also characterized the protein as an RNA  polymerase II elongation factor. |
| ubiquitin carboxyl-terminal esterase L1 (ubiquitin thiolesterase) | 21361091 | Ubiquitin thiolesterase activity; ubiquitin-dependent protein catabolism, predicted. |
| ubiquitin protein ligase E3B isoform a | 35493952 | The modification of proteins with ubiquitin is an important cellular mechanism for targeting abnormal or short-lived proteins for degradation. Ubiquitination involves at least three classes of enzymes: ubiquitin-activating enzymes, or E1s, ubiquitin-conjugating enzymes, or E2s, and ubiquitin-protein ligases, or E3s. This gene encodes a member of the E3 ubiquitin-conjugating enzyme family. The encoded protein may interact with other proteins and play a role in stress response. |
| ***Signal Transduction & Membranes* (34%)** |  |  |
| acetylserotonin O-methyltransferase-like | 4757794 | Transferase activity, predicted. |
| alpha-2-HS-glycoprotein (AHSG) | 4502005 | Promotes endocytosis, possesses opsonic properties and influences the mineral phase of bone. Shows affinity for calcium and barium ions. |
| annexin A2 isoform 2 | 4757756 | Calcium-regulated membrane-binding protein whose affinity for calcium is greatly enhanced by anionic phospholipids. It binds two calcium ions with high affinity. |
| bromodomain and PHD finger containing, 3 | 20555413 |  |
| cell division cycle 25C protein isoform b | 12408658 | Plays a key role in the regulation of cell division. The encoded protein is a tyrosine phosphatase and belongs to the Cdc25 phosphatase family. It directs dephosphorylation of cyclin B-bound CDC2 and triggers entry into mitosis. It is also thought to suppress p53-induced growth arrest. |
| cyclin G2 | 4757936 | May play a role in growth regulation and in negative regulation of cell cycle progesssion. |
| cyclin M2 isoform 1 | 40068053 |  |
| cysteine-rich motor neuron 1 | 10092639 | May play a role in CNS development by interacting with growth factors implicated in motor neuron differentiation and survival. May play a role in capillary formation and maintainance during angiogenesis. Modulates BMP activity by affecting its processing and delivery to the cell surface. |
| dishevelled associated activator of morphogenesis 2 | 40548415 |  |
| elongation of very long chain fatty acids  (FEN1/Elo2, SUR4/Elo3, yeast)-like 2 | 40254906 | Cellular component: integral to membrane, predicted. |
| flotillin 2 | 4758394 | May act as a scaffolding protein within caveolar membranes, functionally participating in formation of caveolae or caveolae-like vesicles. May be involved in epidermal cell adhesion and epidermal structure and function. |
| forkhead box P1 isoform 1 | 18644886 | Forkhead box transcription factors play important roles in the regulation of tissue- and cell type-specific gene transcription during both development and adulthood. Forkhead box P1 protein contains both DNA-binding- and protein-protein binding-domains. This gene may act as a tumor suppressor as it is lost in several tumor types and maps to a chromosomal region (3p14.1) reported to contain a tumor suppressor gene(s). |
| GPI anchored molecule like protein | 4504033 | May play a role in the apoptotic pathway or cell-cycle regulation induced by p53 after DNA damage. |
| heterogeneous nuclear ribonucleoprotein A2/B1 isoform B1 | 14043072 | Involved with pre-mRNA processing. Forms complexes (ribonucleosomes) with at least 20 other different hnRNP and heterogeneous nuclear RNA in the nucleous. |
| heterogeneous nuclear ribonucleoprotein F | 4826760 | This protein is a component of the heterogenous nuclear ribonucleoprotein (hnRNP) complexes which provide the substrate for the processing events that pre-mRNAs undergo before becoming functional, translatable mRNAs in the cytoplasm. Probably binds G-rich sequences in pre-mRNAs. |
| heterogeneous nuclear ribonucleoprotein K isoform b | 14165435 | One of the major pre-mRNA-binding proteins. Binds tenaciously to poly(C) sequences. Likely to play a role in the nuclear metabolism of hnRNAs, particularly for pre-mRNAs that contain cytidine-rich sequences. Can also bind poly(C) single-stranded DNA. |
| membrane-spanning proteoglycan NG2, similar to | 51464985 | Proteoglycan playing a role in cell proliferation and migration which stimulates endothelial cell motility during microvascular morphogenesis. May also inhibit neurite outgrowth and growth cone collapse during axon regeneration. Cell surface receptor for collagen alpha 2(VI) which may confer cells ability to migrate on that substrate. Binds through its extracellular N-terminus to growth factors and extracellular matrix proteases, modulating their activity. May regulate MPP16-dependent degradation and invasion of type I collagen, contributing to melanoma cells invasion. May modulate the plasminogen system by enhancing plasminogen activation and inhibiting angiostatin. Functions also as a signal transducing protein by binding through its cytoplasmic C-terminus scaffolding and signaling proteins. May promote retraction fiber formation and cell polarization through Rho GTPase activation. May stimulate alpha-4, beta-1 integrin-mediated adhesion and spreading by recruiting and activating a signaling cascade through CDC42, ACK1 and BCAR1. May activate FAK and ERK1/ERK2 signaling cascades. |
| Nuclear protein SkiP (Ski-interacting protein) (SNW1 protein) (Nuclear receptor coactivator NCoA-62), similar to | 51458657 | Involved in vitamin D-mediated transcription. Can function as a splicing factor in pre-mRNA splicing. |
| nuclear receptor coactivator 5 | 15147335 | Nuclear receptor coregulator that can have both coactivator and corepressor functions. Interacts with nuclear receptors for steroids (ESR1 and ESR2) independently of the steroid binding domain (AF-2) of the ESR receptors, and with the orphan nuclear receptor NR1D2. Involved in the coactivation of nuclear steroid receptors (ER) as well as the corepression of MYC/c-myc in response to 17-beta-estradiol (E2). |
| nucleophosmin 1 isoform 1 | 10835063 | Associated with nucleolar ribonucleoprotein structures and binds single-stranded nucleic acids. It may function in the assembly and/or transport of the ribosome. |
| polo-like kinase 3 | 41872374 | Serine/threonine protein kinase involved in regulating M phase functions during the cell cycle. May also be part of the signaling network controlling cellular adhesion. In vitro, is able to phosphorylate CDC25C and casein. |
| Ras-GTPase-activating protein SH3-domain-binding protein | 38327552 |  |
| regulator of G-protein signalling 3 isoform 1 | 18644736 | Down-regulates signaling from heterotrimeric G-proteins by increasing the GTPase activity of the alpha subunits, thereby driving them into their inactive GDP-bound form. Down-regulates G-protein-mediated release of inositol phosphates and activation of MAPKs. |
| retinoblastoma binding protein 7 | 4506439 | This protein is a ubiquitously expressed nuclear protein and belongs to a highly conserved subfamily of WD-repeat proteins. It is found among several proteins that binds directly to retinoblastoma protein, which regulates cell proliferation. The encoded protein is found in many histone deacetylase complexes, including mSin3 co-repressor complex. It is also present in protein complexes involved in chromatin assembly. This protein can interact with BRCA1 tumor-suppressor gene and may have a role in the regulation of cell proliferation and differentiation. |
| Rho GDP dissociation inhibitor (GDI) alpha | 4757768 | Rho GDP-dissociation inhibitor activity. |
| SEC13-like 1 isoform b | 34335134 | Has similarity to the yeast SEC13 protein, which is required for vesicle biogenesis from endoplasmic reticulum during the transport of proteins. |
| Sedlin, similar to (Trafficking protein particle complex protein 2) (MBP-1 interacting protein-2A) (MIP-2A) | 51477744 | Binds to MBP1, prevents MBP1-mediated transcriptional repression and antagonizes MBP1-mediated cell death. May play a role in vesicular transport from endoplasmic reticulum to Golgi. |
| sex comb on midleg-like 1 | 5803159 | Putative Polycomb group (PcG) protein. PcG proteins act by forming multiprotein complexes, which are required to maintain the transcriptionally repressive state of homeotic genes throughout development. |
| stromal interaction molecule 2 | 41349446 | Cell membrane single-pass type I membrane protein, possible adhesion molecule with a role in early hematopoiesis by mediating attachment to stromal cells. Influences the survival and/or proliferation of B-cell precursors. |
| SWI/SNF-related matrix-associated actin-dependent regulator of chromatin a4 | 21071056 | Transcriptional coactivator cooperating with nuclear hormone receptors to potentiate transcriptional activation. |
| transcription factor 20 isoform 2 | 31652242 | Transcriptional activator that binds to the regulatory region of MMP3 and thereby controls stromelysin expression. It stimulates the activity of various transcriptional activators such as JUN, SP1, PAX6 and ETS1, suggesting a function as a coactivator. |
| transformation/transcription domain-associated protein | 4507691 | Adapter protein, which is found in various multiprotein chromatin complexes with histone acetyltransferase activity (HAT), which gives a specific tag for epigenetic transcription activation. Plays a central role in MYC (c-Myc) transcription activation, and also participates in cell transformation by MYC. Required for p53/TP53-, E2F1- and E2F4-mediated transcription activation. Also involved in transcription activation mediated by the adenovirus E1A, a viral oncoprotein that deregulates transcription of key genes. Probably acts by linking transcription factors such as E1A, MYC or E2F1 to HAT complexes such as STAGA thereby allowing transcription activation. May be required for the mitotic checkpoint and normal cell cycle progression. |
| zinc finger, CCHC domain containing 11 | 41107445 |  |
| zinc finger protein 37 homolog | 4507963 | May be involved in transcriptional regulation. |
| zinc finger protein 185 (LIM domain) | 6005972 | May be involved in the regulation of cellular proliferation and/or differentiation. |
| zinc finger protein 446 | 8923582 | May be involved in transcriptional regulation. |
| ***Various* (15%)** |  |  |
| 33 kDa protein, similar to | 51465630 |  |
| 40S ribosomal protein SA (p40) (34/67 kDa laminin receptor) (Colon carcinoma laminin-binding protein) (NEM/1CHD4) (Multidrug resistance-associated protein MGr1-Ag), similar to | 41201737 | Laminin receptor activity; cell adhesion, predicted. |
| albumin precursor | 4502027 | Serum albumin, the main protein of plasma, has a good binding capacity for water, Ca(2+), Na(+), K(+), fatty acids, hormones, bilirubin and drugs. Its main function is regulating the colloidal osmotic pressure of the blood. |
| heme oxygenase (decyclizing) 2 | 8051608 | Heme oxygenase cleaves the heme ring at the alpha methene bridge to form biliverdin. Biliverdin is subsequently converted to bilirubin by biliverdin reductase. Under physiological conditions, the activity of heme oxygenase is highest in the spleen, where senescent erythrocytes are sequestrated and destroyed. [CATALYTIC ACTIVITY](http://www.expasy.org/sprot/userman.html#CC_line): Heme + 3 AH_2_ + 3 O_2_ = biliverdin + Fe^2+^ + CO + 3 A + 3 H_2_O. |
| human immunodeficiency virus type I enhancer binding protein 2 | 19923374 | This protein specifically binds to the DNA sequence 5'-GGGACTTTCC-3' which is found in the enhancer elements of numerous viral promoters such as those of SV40, CMV, or HIV1. In addition, related sequences are found in the enhancer elements of a number of cellular promoters, including those of the class I MHC, interleukin-2 receptor, somatostatin receptor II, and interferon-beta genes. It may act in T-cell activation. |
| KIAA0020 isoform 2 | 33620773 | [Synonyms](http://www.expasy.org/sprot/userman.html#DE_line): HBV X-transactivated gene 5 protein, Minor histocompatibility antigen HA-8, HLA-HA8. |
| Laminin receptor 1, similar to | 51467222 | Small ribosomal subunit; receptor activity; structural constituent of ribosome; protein biosynthesis, predicted. |
| mirror-image polydactyly 1 | 23503325 |  |
| mitochondrial ribosomal protein L3 | 6005862 | Mammalian mitochondrial ribosomal proteins help in protein synthesis within the mitochondrion. |
| NudC domain containing 3 | 13357210 |  |
| poly(rC)-binding protein 2 isoform b | 14141166 | Nucleic acid binding, predicted. |
| ribosomal protein S15a, similar to | 51464294 | Structural constituent of the ribosome; protein biosynthesis, predicted. |
| SMC5 protein | 24850456 | Chromosome organization and biogenesis, predicted. |
| step II splicing factor SLU7 | 27477111 | Pre-mRNA splicing occurs in two sequential transesterification steps. This protein is a splicing factor that has been found to be essential during the second catalytic step in the pre-mRNA splicing process. It associates with the spliceosome and contains a zinc knuckle motif that is found in other splicing factors and is involved in protein-nucleic acid and protein-protein interactions. |
| testis specific, 10 | 13376858 |  |
| valosin-containing protein | 6005942 | Valosin-containing protein (VCP) is a member of a family that includes putative ATP-binding proteins involved in vesicle transport and fusion, 26S proteasome function, and assembly of peroxisomes. VCP, as a structural protein, is associated with clathrin, and heat-shock protein Hsc70, to form a complex. VCP has been implicated in a number of cellular events that are regulated during mitosis, including homotypic membrane fusion, spindle pole body function, and ubiquitin-dependent protein degradation. |
